# Mutanobactin-D, a *Streptococcus mutans* Non-Ribosomal Cyclic Lipopeptide, Induces Osteogenic/Odontogenic Differentiation of Human Dental Pulp Stem Cells and Human Bone Marrow Stem Cells

**DOI:** 10.3390/ijms26031144

**Published:** 2025-01-28

**Authors:** Sandra Nikolic, Giuseppe Alastra, Felix Pultar, Lukas Lüthy, Bernd Stadlinger, Erick M. Carreira, Isaac Maximiliano Bugueno, Thimios A. Mitsiadis

**Affiliations:** 1 Orofacial Development and Regeneration, Institute of Oral Biology, Faculty of Medicine, Centre of Dental Medicine, University of Zurich, 8032 Zurich, Switzerland; sandra.nikolic@uzh.ch (S.N.); giuseppe.alastra2@unibo.it (G.A.); 2 Department of Veterinary Medical Sciences, University of Bologna, 40126 Bologna, Italy; 3 Laboratory of Organic Chemistry, Department of Chemistry and Applied Biosciences, ETH Zürich, 8093 Zürich, Switzerland; felix.pultar@phys.chem.ethz.ch (F.P.); lukas.luethy@org.chem.ethz.ch (L.L.); erickm.carreira@org.chem.ethz.ch (E.M.C.); 4 Clinic of Cranio-Maxillofacial and Oral Surgery, University of Zurich, 8032 Zurich, Switzerland; bernd.stadlinger@zzm.uzh.ch; 5 Foundation for Research and Technology—Hellas (FORTH), University of Crete, 700 13 Heraklion, Greece

**Keywords:** human dental pulp stem cells, human bone marrow stem cells, odontogenic differentiation, osteogenic differentiation, mutanobactin-D (Mub-D), *Streptococcus mutans*, tissue regeneration

## Abstract

Bacterium-triggered carious lesions implicate dental hard tissue destruction and the simultaneous initiation of regenerative events comprising dental stem cell activation. *Streptococcus mutans* (*S. mutans*) is a prominent pathogen of the oral cavity and the principal cause of caries. *S. mutans* generates complex products involved in interbacterial interactions, including Mutanobactin-D (Mub-D), which belongs to a group of non-ribosomal cyclic lipopeptides. In the present study, we aimed to analyse the potential role of the synthetic Mub-D peptide in cell populations involved in tissue regenerative processes. To this end, we assessed the *in vitro* effects of Mub-D in human dental pulp stem cells (hDPSCs) and human bone marrow stem cells (hBMSCs). Our data demonstrated a concentration-dependent effect of Mub-D on their viability and a significant increase in their proliferation and osteogenic/odontogenic differentiation. These events were associated with specific changes in gene expression, where *CCDN-1*, *RUNX-2*, *OSX*, *OCN*, *DMP-1*, *DSPP*, and *BMP-2* genes were upregulated. The ability of Mub-D to modulate the osteogenic/odontogenic differentiation of both hDPSCs and hBMSCs and considerably enhance mineralisation in a controlled and concentration-dependent manner opens new perspectives for stem cell-based regenerative approaches in the clinics.

## 1. Introduction

The microorganisms of the oral cavity form a heterogeneous ecological biosystem, which is important in maintaining oral and general health [1,2,3]. An imbalance of the oral environment due to microbial flora alterations affects the dental biofilm composition, which often leads to caries [4,5]. Dental biofilm is formed by oral microorganisms on the tooth enamel surface, where complex interactions between acid-producing bacteria and fermentable carbohydrates occur over time. The anaerobic bacterium *Streptococcus mutans* (*S. mutans*) is an early coloniser of the dental biofilm and the principal cause of dental caries. The successful colonisation of *S. mutans* is due to the production of bacteriocins and the formation of an acid-resistant biofilm. *S. mutans* produces a group of four non-ribosomal cyclic lipopeptides called Mutanobactins (Mub-A, -B, -C, and -D). Mutanobactins might play a role in bacterial interspecies communications within the dental biofilm and, therefore, could affect the initiation and progression of caries [6,7].

Although there is good information concerning Mutanobactins’ structure and interactions with other microorganisms [8,9,10], nothing is known yet about their effects and functions on dental tissues during cariogenesis and regeneration. Carious lesions start with dental hard tissue (i.e., enamel and dentine) demineralisation and their progressive destruction and continue with the inflammation and harm of the connective dental pulp tissue. Dental decay also activates regenerative mechanisms within the dental pulp to repair damaged or lost tissues. The capacity of the dental pulp to repair injuries is fundamental for maintaining tooth integrity and homeostasis. Cell proliferation and dentine secretion via odontoblasts are reduced in the healthy dental pulp tissue, while these processes are enhanced after carious lesions [11,12,13,14]. These cellular responses aim to prevent bacterial invasion into the pulp chamber, restrain the lesion, and activate wound healing. In slow-progressing caries, the activity of the odontoblasts increases, leading to tertiary dentine deposition beneath the bacterial invasion front [11,15]. The increased dentine synthesis is an important process that safeguards the integrity of the odontoblasts and dental pulp tissue. However, fast-progressing caries lead to odontoblast apoptosis, the recruitment of dental pulp stem cells to the caries sites, and their differentiation into a new generation of odontoblasts that form the tertiary dentine [11,16]. Therefore, dental tissue regeneration is ensured by various stem cell populations residing in specific anatomic locations of the pulp tissue [13,17]. Previous studies have shown that human dental pulp stem cells (hDPSCs) can differentiate towards distinctive cell types, including the osteogenic, adipogenic, and neurogenic lineages, and therefore have great clinical potential for cell-based regenerative approaches. Human bone marrow stem cells (hBMSCs) also constitute a source of stem cells for the regeneration of various tissues [18,19,20], including dental tissue regeneration [18,21,22,23]. Previous studies have shown distinctive differentiation potentials for hBMSCs and hDPSCs towards adipogenic, osteogenic, and neurogenic lineages [20,22,23,24]. However, hBMSCs and hDPSCs can differentiate into osteogenic and odontogenic cells [19,25,26,27,28].

The differentiation of stem cells towards distinctive cell types is conditioned by the microenvironment, and therefore, cell-based regenerative treatments also exploit molecules with specific cellular functions able to modulate their differentiation potential [12,21,29,30,31,32]. Consequently, in this study, we assessed the effects of Mub-D on the odontogenic/osteogenic differentiation of in vitro-cultured hDPSCs and hBMSCs. These studies are complemented by gene expression analyses of specific osteogenic/odontogenic markers. Our results show that Mub-D plays an important role in the osteogenic differentiation of hDPSCs and hBMSCs, and we identify Mub-D as a suitable molecule for regenerative applications in clinics.

## 2. Results

### 2.1. Concentration-Dependent Mub-D Effect on hDPSC and hBMSC Viability

**Figure 1:**
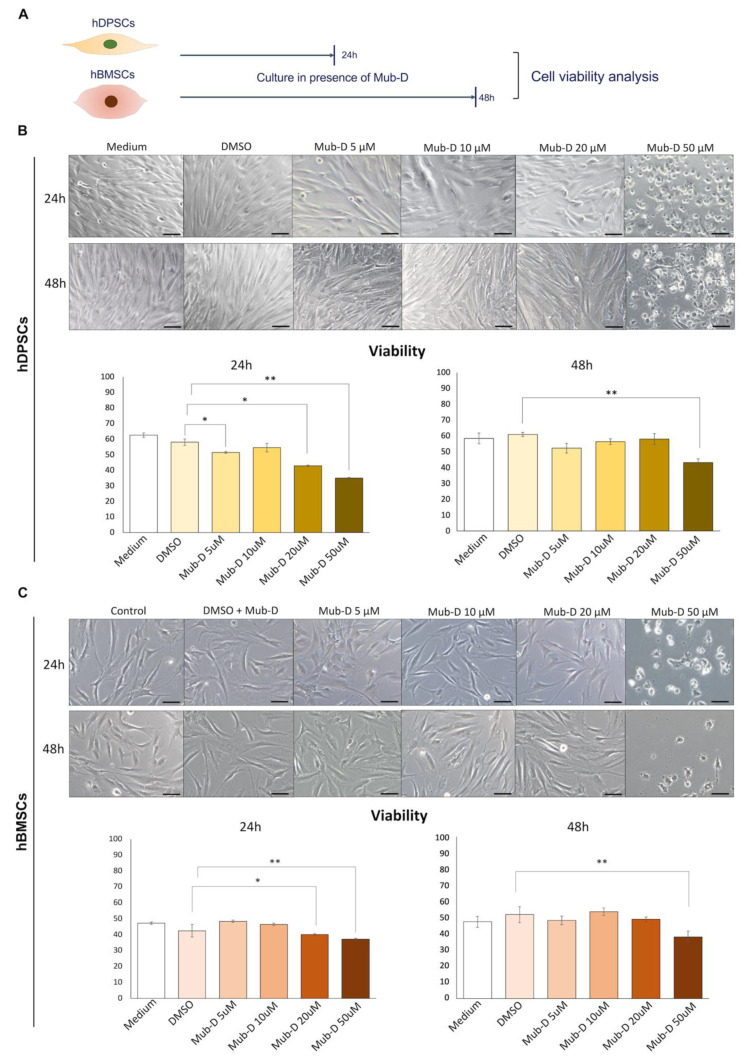
Concentration-dependent effects of Mub-D on hDPSC and hBMSC viability. (**A**) Experimental setup of Alamar Blue (AB) reduction tests for evaluating hDPSC and hBMSC viability in cultures supplemented with Mub-D. Four different concentrations of Mub-D were tested (5, 10, 20, and 50 μM). (**B**,**C**) AB reduction assay was performed in cultures of hDPSCs (**B**) and hBMSCs (**C**) for 24 h and 48 h. Bright-field images of the different culture conditions. Plots present the percentage of AB reduction. Data are presented as average values ± standard deviation. Two-way ANOVA and Tukey post hoc were used to analyse the differences between the experimental groups for each time point. Asterisks represent statistically significant differences between experimental groups for each time point (* *p* value < 0.05; ** *p* value < 0.01). Scale bars: 20 μm.

We cultured hDPSCs and hBMSCs until they reached 60 to 70% confluence, considering that time as T0, and added Mub-D in the culture medium, and then, cultures were processed for either 24 or 48 h (Figure 1A). In both culture periods, the viability of hDPSCs and hBMSCs was reduced in the highest Mub-D concentration (series of 5, 10, 20, and 50 μM). In this study, 10 μM of Mub-D did not significantly affect the viability of both stem cell populations. However, 20 μM of Mub-D had either a moderate or no effect on cell viability. By contrast, 50 μM of Mub-D significantly reduced the viability of hDPSCs and hBMSCs (Figure 1B,C).

**Figure 2:**
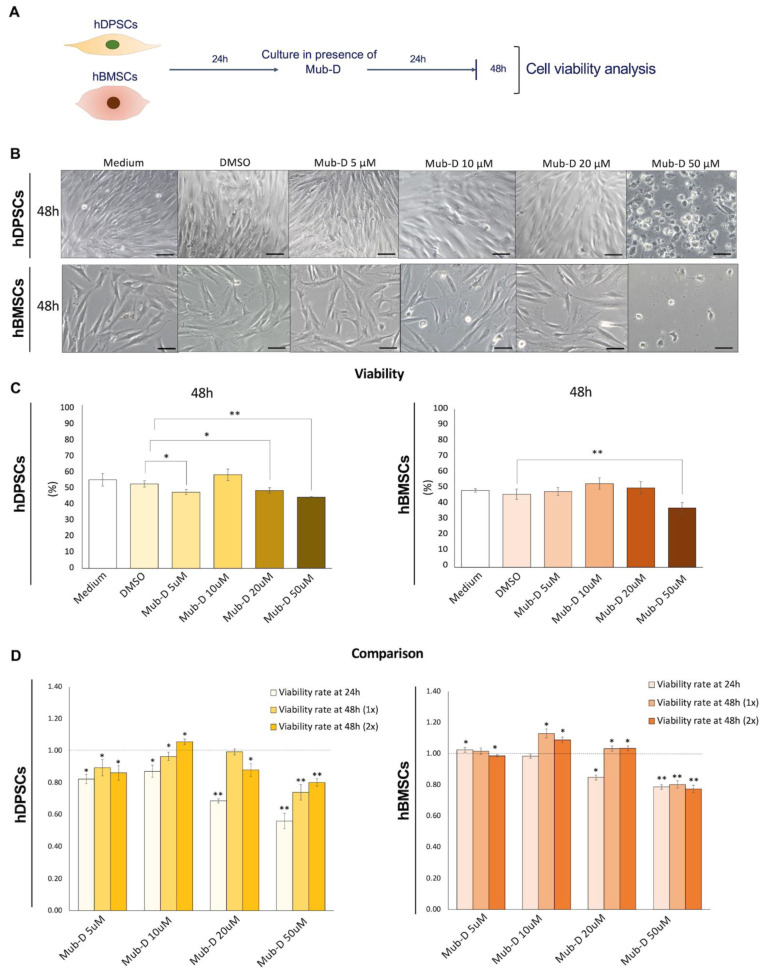
Effects of Mub-D on hDPSC and hBMSC viability. (**A**) Experimental setup of AB reduction test for cell viability evaluation in hDPSCs and hBMSCs after a single or double Mub-D administration within 48h of culture. Four different concentrations of Mub-D were tested (5, 10, 20, and 50 μM). (**B**) Bright-field microscopy images of hDPSCs and hBMSCs after double Mub-D administration. (**C**) AB reduction assay in hDPSC and hBMSC cultures upon Mub-D addition. (**D**) Comparison between the two time points (24 h and 48 h) following a single or double Mub-D administration in cultured hDPSCs and hBMSCs. Data are presented as average values ± standard deviation. Two-way ANOVA and Tukey post hoc were used to analyse the differences between the experimental groups for each time point. Asterisks represent statistically significant differences between experimental groups for each time point (* *p* value < 0.05; ** *p* value < 0.01). Scale bars: 20 μm.

**Figure 3:**
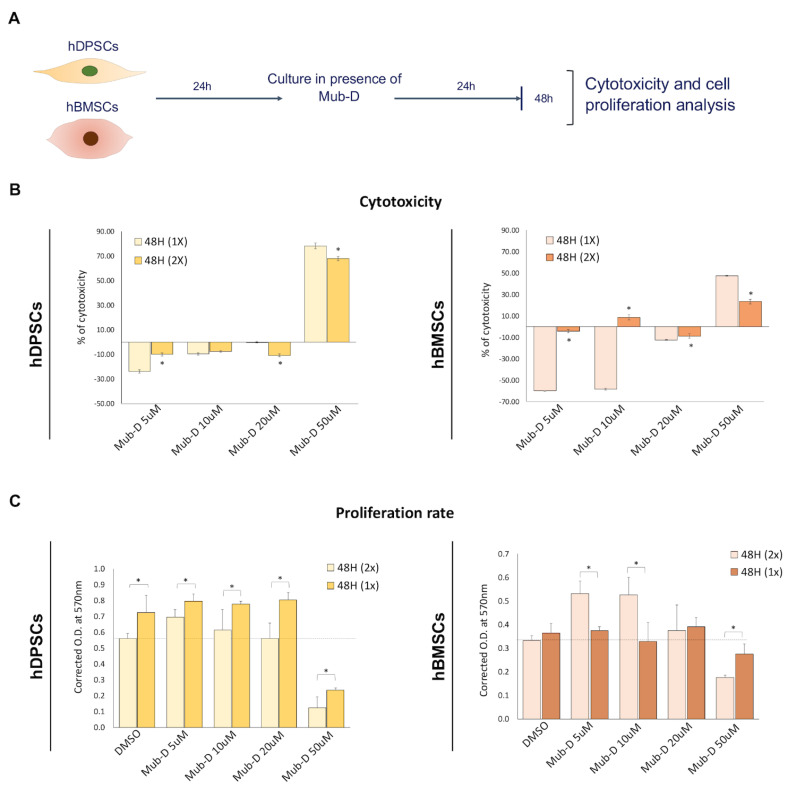
Cytotoxic and proliferative effects of Mub-D in hDPSCs and hBMSCs. (**A**) Experimental setup of crystal violet assays for the evaluation of the cytotoxicity of Mub-D (single or double administration) and its proliferation effects on cultured for 48 h hDPSCs and hBMSCs. Four different Mub-D concentrations were tested (5, 10, 20, and 50 μM). (**B**) Cytotoxicity calculated by crystal violet assay in hDPSC and hBMSC cultures upon Mub-D addition. (**C**) Proliferation of hDPSCs and hBMSCs quantified via crystal violet assay upon Mub-D addition. Comparison between a single or double Mub-D administration in cultured hDPSCs and hBMSCs. Data are presented as average values ± standard deviation. Asterisks represent statistically significant differences between experimental groups for each time point (* *p* value < 0.05).

In another set of experiments, the culture medium of hDPSCs and hBMSCs was supplemented twice with Mub-D at T0 and 24 h, and their viability was analysed after 48 h of culture (Figure 2B,C). A stronger effect compared to the previous experiment was observed in hDPSC and hBMSC viability after adding Mub-D twice in the culture medium (Figure 2C,D). Since 50 μM of Mub-D significantly decreased hDPSC and hBMSC viability, with a 20% to 50% range of cell number reduction (Figure 2D), we can assume that this concentration is toxic for the cells. Crystal violet assays allowed the evaluation ofMub-D cytotoxicity in both cell populations (Figure 3A). At 50 μM of Mub-D, there was 67% to 78% cytotoxicity for hDPSCs and 23% to 47% for hBMSCs (Figure 3B). No cytotoxic effects were observed for other Mub-D concentrations (i.e., 5, 10, or 20 μM) (Figure 3B).

### 2.2. Mub-D Effect on hDPSC and hBMSC Proliferation

Mub-D addition in the culture medium at concentrations of 5, 10, and 20 μM increased the proliferation rate of hDPSCs and hBMSCs (Figure 3C). The most pronounced increase in hDPSC proliferation was observed when the culture medium was supplemented once and not twice with Mub-D (Figure 3C). By contrast, the proliferation rate of hBMSCs after 48 h of culture was more prominent after the double addition of Mub-D (Figure 3C).

**Figure 4:**
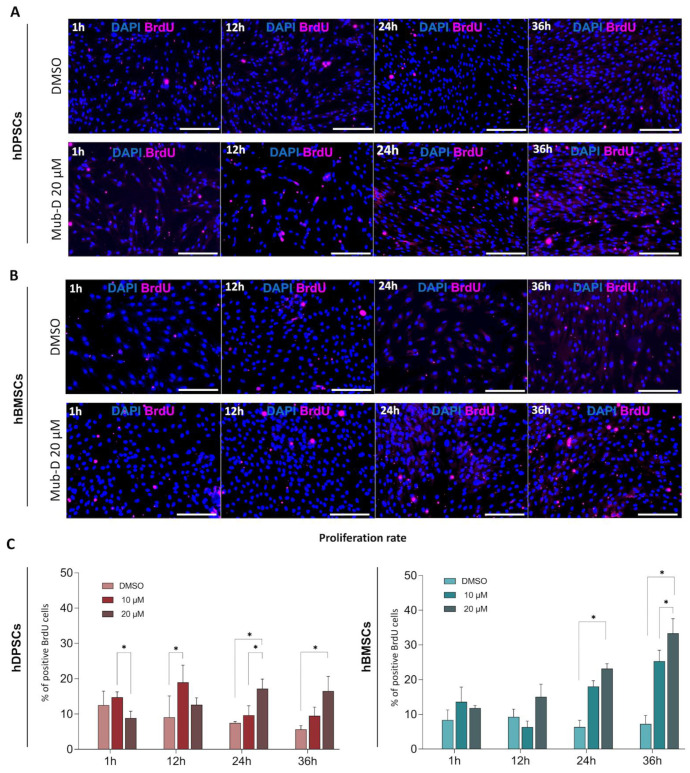
Effects of Mub-D on hDPSC and hBMSC proliferation. Cell proliferation visualised upon BrdU immunostaining. (**A**,**B**) Inverted fluorescence microscopy images showing BrdU-positive hDPSCs (**A**) and hBMSCs (**B**) cultured in the presence of 20 μM Mub-D in DMSO. Four time points were analysed (1, 12, 24, and 36 h). (**C**) Plots present the percentage of BrdU-positive cells in hDPSCs and hBMSCs cultures after Mub-D administration. Two concentrations of Mub-D (10 and 20 μM) were analysed. Data are presented as average values ± standard deviation. Two-way ANOVA and Tukey post hoc tests were used to compare time points for each cell type. Asterisks represent statistically significant differences between experimental groups for each time point (* *p* value < 0.05). Scale bar: 100 μm.

To complement the cell proliferation assay, we added 5-bromo-2′-deoxyuridine (BrdU) to the medium of hDPSCs and hBMSCs cultured with Mub-D. The cells were fixed after 1 h, as well as at 12, 24, and 36 h, of culture. Following culture, we conducted BrdU immunostaining to quantify the number of cells entering the S-phase (Figure 4). An increased number of hDPSCs (Figure 4A,C) and hBMSCs (Figure 4B,C) in the S-phase was observed upon the addition of 20 μM Mub-D compared to DMSO addition. Comparing the enhanced proliferation rates between these two stem cell populations revealed that hBMSCs are more prone to proliferation than hDPSCs under the influence of Mub-D. This stands true for both culture periods: 24 h (hDPSCs = average 17.21% ± 2.71 standard deviation; hBMSCs = average 23.18% ± 1.37 standard deviation) and 36 h (hDPSCs = average 16.56% ± 4.14 standard deviation; hBMSCs = average 33.34% ± 4.23 standard deviation) (Figure 4C). A stronger proliferative effect was noted with the addition of 20 μM compared to 10 μM of Mub-D, as quantified via the two-way ANOVA and Tukey post hoc test (20 μM Mub-D, *p*-value = 0.047; 10 μM Mub-D, *p*-value = 0.035) (Figure 4C).

### 2.3. Osteogenic/Odontogenic Effect of Mub-D in hDPSCs and hBMSCs

**Figure 5:**
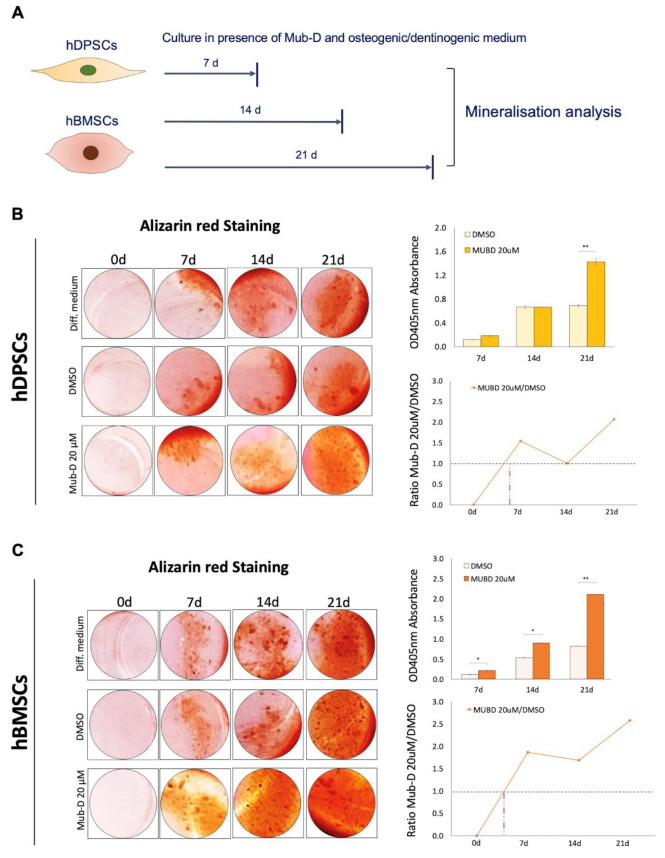
Effects of Mub-D on hDPSC and hBMSC mineral deposition. (**A**) The experimental setup for the Alizarin red staining (ARS) assay was used to evaluate mineralisation in cultures of hDPSCs and hBMSCs supplemented with 20 μM Mub-D for 7, 14, and 21 days. (**B**,**C**) Bright-field images of ARS on cultured hDPSCs (**B**) and hBMSCs (**C**) in the presence of 20 μM Mub-D, DMSO, or odontogenic/osteogenic medium alone. The right-side plots present the mineralisation rates between hDPSCs (**B**) and hBMSCs (**C**) cultured with Mub-D and DMSO. Data are presented as average values ± standard deviation. Asterisks indicate statistically significant differences between experimental groups for each time point (* *p* value < 0.05; ** *p* value < 0.01).

hDPSCs and hBMSCs have a great potential to differentiate in vitro into odontoblasts and osteoblasts, respectively, to secrete specific matrices for dentine and bone formation and to induce their mineralisation [23,33]. The osteogenic/odontogenic culture medium of hDPSCs and hBMSCs was supplemented with 20 μM Mub-D for 7, 14, and 21 days (Figure 5A). The medium was changed every two days. Alizarin Red staining was performed upon culture to evaluate Mub-D effects on mineralisation (Figure 5B,C). We found that Mub-D significantly increased the mineralisation rate in both hDPSC (Figure 5B) and hBMSC (Figure 5C) cultures compared to cells cultured with DMSO or osteogenic/odontogenic medium only. Mub-D addition in the culture medium considerably accelerated the mineralisation process, showing a two-time increase in the deposition of minerals by hDPSCs cultured for 21 days (Figure 5B) and a three-time increase in hBMSC cultures (Figure 5C), compared to DMSO cultures.

### 2.4. Effect of Mub-D on Gene Expression in hDPSCs and hBMSCs

**Figure 6:**
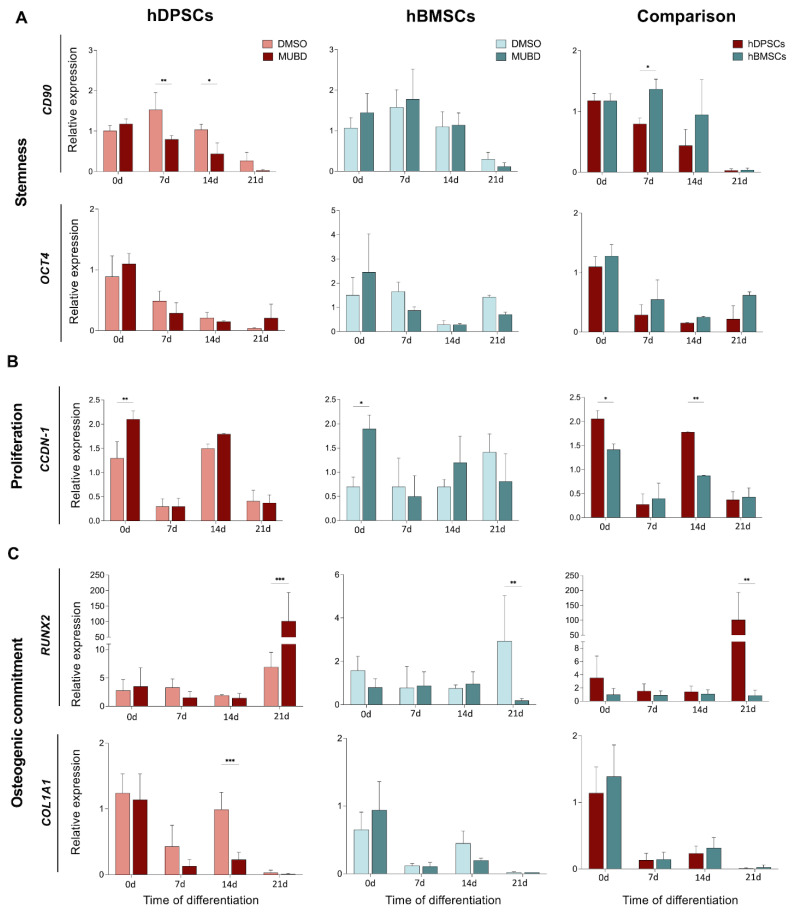
Effects of Mub-D on gene expression in hDPSCs and hBMSCs. (**A**–**C**) *CD90, OCT4* (**A**), *CCDN1* (**B**), *RUNX2*, and *COLIA1* expression in hDPSCs and hBMSCs cultured in the presence of Mub-D for 0, 7, 14, and 21 days in odontogenic/osteogenic medium. The relative expression value on comparison graphs is normalised for each gene relative to Mub-D-treated hDPSCs at culture day 0. Statistical analysis data are presented as average values ± standard deviation. Two-way ANOVA and Tukey post hoc tests were used to compare hDPSCs and hBMSCs and time points for each cell type. Asterisks represent statistically significant differences between distinctive time points and day 0 as control (* *p* value < 0.05; ** *p* value < 0.01; *** = *p* value < 0.001).

We next assessed the expression of stem cell-specific genes *CD90* [34] and *OCT4* [35,36] and the cell cycle progression gene *CCDN-1* [37] in hDPSCs and hBMSCs cultured in the odontogenic/osteogenic medium in the presence of 20 μM Mub-D (Figure 6A–C). Mub-D addition in the culture medium of hDPSCs rapidly downregulated *CD90* expression. By contrast, *CD90* downregulation in hBMSCs was not as obvious as for hDPSCs (Figure 6A). A continuous decrease in *OCT4* expression was observed in untreated and Mub-D-treated hDPSCs and hBMSCs after 21 days of differentiation, demonstrating that Mub-D addition does not significantly modulate *OCT4* expression under these culture conditions (Figure 6A). Similarly, in the same cultures, *CD90* expression was progressively reduced in both stem cell populations. Mub-D moderately upregulated *CCDN1* expression in both stem cell populations at early culture stages, but its expression was downregulated after 21 days of culture (Figure 6B).

**Figure 7:**
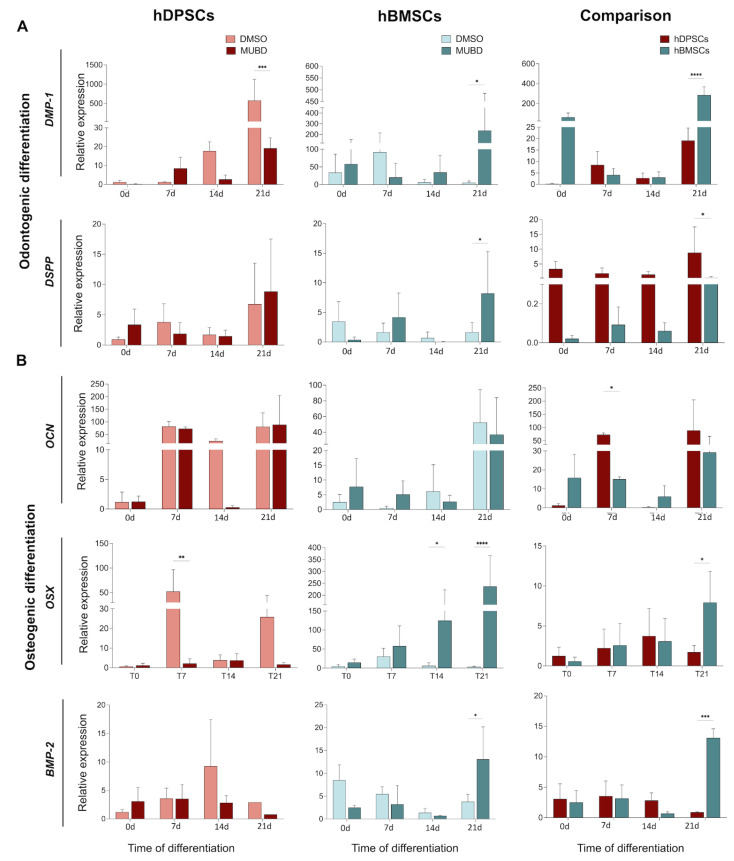
Effect of Mub-D on odontogenic/osteogenic genes expression in hDPSCs and hBMSCs. (**A**,**B**) *DMP-1* and *DSPP* (**A**) and *OCN*, *OSX*, and *BMP2* (**B**) relative mRNA expression in hDPSCs and hBMSCs cultured in the presence of Mub-D at 0, 7, 14, and 21 days. The relative expression value on comparison graphs is normalised for each gene relative to hDPSCs treated with Mub-D at T0. Statistical analysis data are presented as average values ± standard deviation. Two-way ANOVA tests, followed by the Tukey post hoc test, were used to compare hDPSCs and hBMSCs and time points for each cell type. Asterisks represent statistically significant differences between various time points and day 0 as control (* *p* value < 0.05; ** *p* value < 0.01; *** = *p* value < 0.001; **** *p* value < 0.0001).

We further investigated the expression of osteogenic/odontogenic genes *RUNX2* [38,39], *COL1A1* (collagen type I-A1 domain) [33,40] (Figure 6C), *DMP-1* (dentin matrix acidic phosphoprotein 1) [41,42], *DSPP* (dentine sialophosphoprotein) [42,43] (Figure 7A), *OSX* (osterix) [44,45], *OCN* (osteocalcin) [46,47], and *BMP2* (bone morphogenetic protein 2) [48,49] (Figure 7B) in cultured hDPSCs and hBMSCs in the presence of Mub-D. *RUNX2* expression was not affected by Mub-D addition in either hDPSCs or hBMSCs, except in hDPSCs cultured for 21 days where its expression was upregulated (Figure 6C). By contrast, in hBMSCs cultured for 21 days, *RUNX2* expression was downregulated compared to DMSO cultures (Figure 6C). *COLIA1* expression was not significantly affected by Mub-D in hDPSCs and hBMSCs cultured for 21 days, except in hDPSCs cultured for 14 days where its expression was downregulated compared to DMSO cultures (Figure 6C). The expression of *DMP-1* and *DSPP* was upregulated in hBMSCs cultured for 21 days (Figure 7A). By contrast, *DMP-1* expression was downregulated, and *DSPP* expression was not significantly affected in hDPSCs cultured for 21 days (Figure 7A). *OCN* expression was upregulated during osteogenic/odontogenic differentiation in both hDPSCs and hBMSCs, but Mub-D did not affect its expression. *OSX* and *BMP-2* expression was strongly upregulated in hBMSCs cultured with Mub-D for 21 days (Figure 7B). By contrast, in hDPSCs cultured for 21 days, *OSX* and *BMP-2* expression was not affected upon Mub-D addition (Figure 7B).

## 3. Discussion

Regenerative medicine aims to repair or replace damaged tissues and offers alternative therapeutic practices to currently used clinical methods [50,51,52,53]. The translation of experimental regenerative approaches to patients is a big challenge, intending to advance medicine by tackling still unmet healthcare needs [31,54,55,56]. Regenerative therapies involve a variety of stem cell populations and specific peptides to promote repair and restore the structure and functions of the damaged tissues [31,52,57]. Mesenchymal stem cells have gained great attention due to their multilineage differentiation potential and immunomodulatory properties, important features for the effectiveness of regenerative treatments [31,53,58].

Innovative regenerative approaches based on mesenchymal stem cells and specific molecules could also be used in dental clinics to restore the physiology and structural integrity of damaged dental tissues [51,52,53,58,59]. Previous in vitro and in vivo studies have demonstrated the potential of human dental pulp stem cells (hDPSCs) for tissue regeneration in patients and experimental animal models [32,60,61]. Although the efficient use of specific peptides constitutes an important parameter for successful regenerative treatments, our actual knowledge about their action in the various stem cell lines is ambiguous [50,51]. Indeed, most information is based on in vitro experimental setups that certainly do not reflect in vivo conditions [31,56,62]. Only several molecules have already been used in clinics to control stem cell fate and behaviour in pathological conditions [63,64,65,66,67]. However, new disease-specific molecules with promising regenerative outcomes continuously emerge since all these regenerative treatments are in their infancy. Synthetic peptides started to be used as biological tools to modulate and enhance bone or dental regeneration [29,68,69]. These peptides mimic the structure of natural molecules and might induce and enhance mineralisation [29,70,71,72]. The functions of only a few of these novel synthetic peptides have been assessed, both in vitro and in vivo, with encouraging outcomes. For example, PTH1-34 and PepGen P-15 successfully repaired bone abnormalities and could thus be proposed for preclinical or clinical trials [72,73,74,75,76].

*Streptococcus mutans* (*S. mutans*) produces Mutanobactins, a group of four non-ribosomal cyclic lipopeptides (Mub-A, -B, -C, and -D), which could affect the caries outcome [6,7,8,10]. This outcome also depends on dental pulp stem cells (DPSCs) and their capacity to regenerate damaged dental tissues. Carious lesions often induce odontoblast apoptosis and activate complex regenerative mechanisms within the dental pulp [77,78,79]. Under these conditions, dental pulp stem cells are activated to replace the dying cells and ensure dental tissue repair. The dental pulp stem cells differentiate into odontoblast-like cells, which produce the reparative dentine that protects dental pulp tissue integrity by slowing down bacterial invasion [80,81]. Human DPSCs (hDPSCs) are characterised by the expression of several mesenchymal stem cell markers (CD29, CD73, CD105, and CD44) and extracellular matrix molecules such as collagen, vimentin, laminin, and fibronectin [13,17,80,81,82,83]. hDPSCs possess great regenerative potential since they are multipotent and can differentiate into various cell types, including adipocytes, chondrocytes, osteoblasts, and odontoblasts [22,25,84,85]. Therefore, hDPSCs are attractive for tissue engineering purposes, especially when used as autologous transplants for tissue regeneration [58,61]. hDPSCs reside in one or more distinct storage site(s), also called stem cell niches [17,83,86,87,88]. Niche-derived and external signals both influence stem cell fates and functions. Previous findings have shown that several molecules, such as bone morphogenetic proteins (BMPs) and Wnts, Nogo-A, and Notch proteins, are important regulators of stem cell function [64,83,85,89,90,91,92,93,94]. Under the influence of these molecules, hDPSCs proliferate and engraft to the carious injury area, where they will finally differentiate into odontoblast-like cells [58,95]. Dental pulp tissue regeneration involves significant molecular changes, including the release of signalling molecules from dentine [77,78,79,95] and the reactivation of Notch signalling in the perivascular stem cell niches [87,96,97]. hDPSCs located at the perivascular niches also participate in tooth repair at various time points [58,87,98,99].

**Figure 8:**
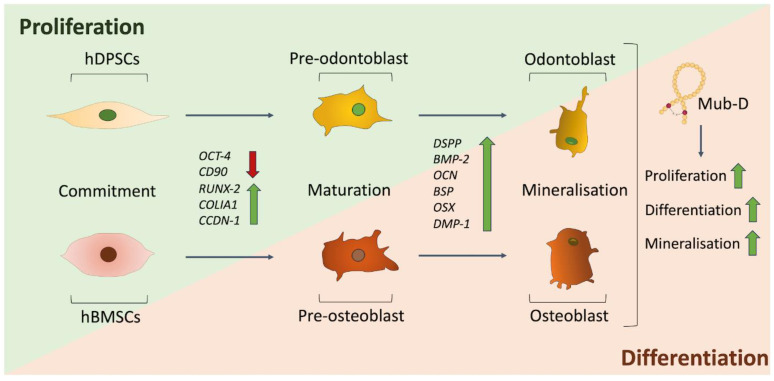
Mub-D induces odontogenic/osteogenic differentiation of hDPSCs and hBMSCs. Schematic representation of the effects of Mub-D in proliferation, differentiation, and mineral deposition of hDPSCs and hBMSCs cultured in odontogenic/osteogenic conditions. The expression of various genes during these processes is indicated. Gene upregulation is indicated by green arrows, and downregulation is indicated by red arrows.

Although the Mub-D peptide was recently synthesised and its structural assignment completed [7], nothing is known about the Mub-D effects on cells involved in dental tissue regeneration upon decay. Our findings highlight significant Mub-D effects in hDPSCs by promoting their odontogenic behaviour and enhancing mineralisation. Similarly, Mub-D stimulates the osteogenic potential of human bone marrow stem cells (hBMSCs) and increases hard tissue formation. Therefore, the function of Mub-D is important in hard tissue formation and, most specifically, during the repair/regeneration of injured dental or bone tissues. A shift in gene expression characterises the fate change of hDPSCs and hBMSCs and their final differentiation into odontoblasts/osteoblasts to form dentine and bone, respectively. The increased expression of odontogenic- and osteogenic-related genes, including *RUNX2*, *DSPP*, *DMP-1*, *OSX,* and *OCN*, accompanies the formation of these two specific mineralised tissues via hDPSCs and hBMSCs. *RUNX2* is a major control gene for mesenchymal stem cell differentiation into preosteoblasts [38,44,100], *OSX* is involved in the transition of preosteoblasts to mature osteoblasts [44,45,101], *DSPP* is essential for odontoblast differentiation and function [43,102,103], and *DMP-1* expression is associated with odontoblast maturation [41,102,104]. The upregulation of these odontogenic/osteogenic genes in both stem cell populations after Mub-D administration correlates with the broad mineral formation visualised by Alizarin red in the in vitro cell cultures. The significantly higher *DSPP* expression in hDPSCs cultured in the presence of Mub-D, compared to hBMSCs, indicates their odontogenic commitment [43,105]. In contrast, the increased expression of *BMP2*, a potent osteogenic factor [48,106], in hBMSCs treated with Mub-D, but not in hDPSCs, aligns with the osteogenic differentiation of hBMSCs and a status favouring odontogenesis rather than osteogenesis in hDPSCs (Figure 8).

Obviously, in vitro differentiation assays do not faithfully mimic the in vivo physiology of tissues and organs [107,108]. Our actual in vitro study lacks dental pulp tissue complexity, consisting, among others, of the absence of the immune, vascular, and neuronal systems that are important for the dynamic microenvironment of stem cells. All these parameters represent relevant limitations of the present findings that could be resolved upon additional in vitro (e.g., “organ-on-chip” technology), ex vivo, and in vivo experimental studies [62,109]. Therefore, any attempts to apply Mub-D in the clinics to promote hard tissue regeneration should be prior validated in various animal models in vivo. The clinical applications should not be limited to dentine repair after carious and traumatic dental lesions. Mub-D could also have a beneficial role in the regeneration of damaged periodontal tissues in the case of severe periodontitis and the repair of the alveolar bone upon either fracture or surgery for dental implant placement.

## 4. Materials and Methods

### 4.1. Collection and Culture of Cells

Experiments were performed at the Centre of Dental Medicine of the University of Zurich and approved by the Ethics Committee of the Canton of Zurich (reference number 2012-0588; last notification from authorities was received on 7 November 2023, ref. number: A230123-00) [20]. Third molars were obtained from anonymous healthy patients between 18 and 35 years of age after their written informed consent. Dentists performed tooth extractions at the oral surgery department. All procedures were implemented following the current guidelines [20,25]. Human dental pulp stem cells (hDPSCs) were isolated from the dental pulp of extracted teeth, as previously described [27,82]. In total, 20 fresh dental pulps were extracted from 12 individuals. Briefly, dental pulps were enzymatically digested for one hour at 37 °C in a solution of collagenase (3 mg/mL; Life Technologies Europe, Zug, Switzerland) and dispase (4 mg/mL; Sigma-Aldrich Chemie GmbH, Buchs, Switzerland). These hDPSCs were tested and found negative for HIV-1, hepatitis B, hepatitis C, mycoplasma, bacteria, yeast, and fungi. A filtered single-cell suspension was plated in a 40 mm Petri dish with an hDPSC growth medium containing modified minimum essential medium α (MEMα; Sigma-Aldrich Chemie GmbH, Buchs, Switzerland) with 10% heat-inactivated foetal bovine serum (FBS; PAN Biotech GmbH, Aidenbach, Germany), 1% penicillin/streptomycin (Sigma-Aldrich Chemie GmbH, Buchs, Switzerland), 1% L-glutamine (Sigma-Aldrich Chemie GmbH, Buchs, SG, Switzerland), and 0.5 μg/mL fungizone (Life Technologies Europe BV, Zug, ZG, Switzerland) after washing away the enzymatic solution. hDPSCs were passaged at 80–90% confluence and expanded in the same growth medium in T75 culture flasks.

Human bone marrow stem cells (hBMSCs) were obtained from Lonza (Basel, Switzerland). These cells were isolated from the bone marrow of posterior iliac crests of adult healthy individuals. hBMSCs were cryopreserved after their second culture passage. A certificate reporting the health status of the purchased cells was provided (negative for HIV-1, hepatitis B, hepatitis C, mycoplasma, bacteria, yeast, and fungi). hBMSCs were cultured and expanded in a manner similar to the hDPSCs medium containing MEMα, 10% FBS, 1% penicillin/streptomycin, 1% L-glutamine, and 0.5 μg/mL fungizone. The medium was changed every 2 days.

### 4.2. Mutanobactin-D Synthesis and Use

Mutanobactin-D (Mub-D) was synthesised as previously described [6,7]. A 20 mM Mub-D stock solution was diluted in dimethyl sulfoxide (DMSO; Sigma-Aldrich Chemie GmbH, Buchs, Switzerland) [7]. While the effects of Mutanobactins (A, B, and D) in biofilms and yeast growth have been evaluated in previous studies [8,9], there is no study available on the Mub-D effects on eukaryotic cells. Therefore, we analysed Mub-D effects on cell viability and proliferation using four different concentrations (5, 10, 20, and 50 μM). To obtain the final concentration of 20 μM for the experiments with hDPSCs and hBMSCs, we used one μL of the stock solution for one mL of odontogenic/osteogenic medium. These cell populations were also treated without Mub-D only in the presence of DMSO (<0.001%, as the vehicle control). The Mub-D effects were compared to the DMSO-treated cells.

### 4.3. Cell Viability

Cell viability was determined using the colourimetric Alamar Blue test (AB; Catalog number DAL1025, Life Technologies Europe BV, Zug, Switzerland). AB contains resazurin, a cell-permeable, non-toxic, and fluorescent blue indicator dye. Through an oxidation–reduction reaction, resazurin undergoes a colourimetric change (the blue colour turns to red colour and becomes fluorescent) in response to cellular metabolic reduction. Briefly, after 24 or 48 h of culture, cells were washed, and 1 mL of 10% AB was diluted in an MEMα medium without phenol red. In total, 100 μL of this AB-containing medium was transferred to 96-well plates (realised in triplicates) and measured at 570 and 600 nm to determine the percentage of Alamar-Blue reduction.

### 4.4. Cell Proliferation Analyses

Crystal violet dye was used to assess the proliferation rate of cells by measuring the total DNA mass of viable and adherent cells. Cells were seeded in 24-well plates and treated with crystal violet dye according to the previously described protocol [110]. Following 48 h of Mub-D treatment, cells were fixed with 4% PFA for 10 min at room temperature (RT), washed with PBS, and incubated with 200 µL of 0.5% crystal violet staining solution (Sigma-Aldrich Chemie GmbH, Buchs, Switzerland) for 20 min at RT on a bench rocker having 20 oscillations/min. Then, cells were washed with tap water and incubated with 200 µL of methanol for 20 min at RT. Finally, 100 µL (in triplicates) was transferred to a 96-well plate, and the optical density was measured at 570 nm (OD570). The average OD570 of non-stimulated cells was set to 100%. The percentage of viable stimulated cells was calculated and corrected with control wells OD570. The cytotoxicity of Mub-D was calculated using the percentage cytotoxicity formula: ((OD570^DMSO^-OD570^MUB-D^)/OD570^DMSO^) × 100%.

We used 5-Bromo-2′-deoxyuridine (BrdU), which is a thymidine analogue that incorporates into nuclear DNA during the S-phase of the cell cycle [111]. For the detection of the proliferating hDPSCs and hBMSCs in the S-phase, we seeded these cells in μ-slide 8-well uncoated plates (ref: 80806, Vitaris AG, Baar, Switzerland) and cultured them in the proper culture medium supplemented with either 10 µM or 20 µM Mub-D. The cells were fixed with 4% PFA for 20 min at RT after 1, 12, 24, and 36 h of culture and then rinsed with 1X PBS, incubated with a citrate buffer at pH6 10mM for 30 min at 80 °C, cooled down for 30 min, rinsed 3 times with 1X PBS, and incubated with HCl 2N for 20 min at RT. Then, cells were washed with PBS and incubated with a borate buffer for 30min at RT, then with a blocking buffer of 0,1% Triton and 10% FBS in 1X PBS for 1 h at RT, and finally with a rabbit BrdU primary antibody 1:200 (Abcam, catalog number: ab6326, Life Technologies Europe BV, Zug, Switzerland) in the blocking buffer solution overnight. Thereafter, cells were rinsed with PBS and incubated with the anti-rat secondary antibody Alexa647 IgG 1:1000 (Invitrogen, ref: A21447, Life Technologies Europe BV, Zug, Switzerland) and DAPI (4′,6-diamidino-2-phenylindole) 1:1000 for 2 h in a blocking buffer at RT. All cell types from the BrdU assay were analysed using a widefield Leica DM6000B microscope with the Leica DFC350FX camera (Leica Microsystems (Schweiz) AG Verkaufsgesellschaft, Heerbrugg, Switzerland) and LAS X software (Leica LAS X 4.4 Falcon FLIM, V4.4.0.24861) using 10× objectives. Single images were merged, and positive cells were counted using FIJI (version 2.9.0).

### 4.5. Cell Differentiation Assays

For cytodifferentiation assays, 80–90% confluent hDPSCs and hBMSCs cultured in the T75 flasks were washed with PBS before their detachment with trypsin-EDTA 0.05% for 3 min at 37 °C. The action of trypsin was blocked by adding five volumes of the culture medium supplemented with 10% FBS. After centrifugation, 2.5 × 10^5^ cells per well were seeded onto 6-well plates (Sarsted, AG, Switzerland) for gene expression analysis. For assessing mineralisation, 5 × 10^4^ cells per well were seeded either onto 24-well plates (Sarsted, AG, Switzerland) or in μ-Slide 8-well uncoated plates (Catalog number: 80806, Vitaris, Baar, Switzerland) for staining and microscopic analyses. All experiments were performed in triplicates.

The two stem cell populations were differentiated into specific cell types using an odontogenic/osteogenic differentiation medium for up to 21 days. The differentiation medium consisted in MEMα with 10% FBS, 1% penicillin/streptomycin, 0.5 μg/mL fungizone, 0.1 μM dexamethasone (Sigma-Aldrich Chemie GmbH, Buchs, Switzerland), 10 mM β-glycerophosphate (Sigma-Aldrich Chemie GmbH, Buchs, Switzerland), 50 μg/mL ascorbic acid (Sigma-Aldrich Chemie GmbH, Buchs, Switzerland), and 10 ng/mL TGF-β1 (Sigma-Aldrich Chemie GmbH, Buchs, Switzerland) at 37 °C in a 5% CO_2_ incubator. Trypsin-EDTA 0.25% was used to harvest the cells.

### 4.6. Mineralisation Assessed via Alizarin Red Staining

The extent of mineral products deposited by the cultured hDPSCs and hBMSCs in the odontogenic/osteogenic medium, either in the presence or absence of Mab-D, was analysed at different time points (0, 7, 14, and 21 days) via Alizarin red staining (catalog number: A5533, Sigma-Aldrich Chemie GmbH, Buchs, Switzerland). Calcium deposits were specifically stained bright orange/red. Cell cultures were washed twice with PBS and fixed with 4% PFA for 10 min at RT. The staining solution was prepared by dissolving 2% Alizarin red in distilled water. Upon fixation, cultures were rinsed with distilled water and incubated with Alizarin red solution for 15 min at RT. Excess Alizarin red was removed with three washes of distilled water. Then, colorimetric detection at 405 nm in a 96-well plate was performed to quantify staining.

### 4.7. RNA Extraction, Reverse Transcription, and Quantitative Real-Time PCR (qRT-PCR)

Cells were cultured in 6-well plates, and total RNA was extracted using TRIzol^TM^ reagent (catalog number: 15596026; ThermoFisher, Zug, Switzerland) following the manufacturer’s instructions. RNA extraction and purification were performed as previously described [20]. Primers for *GAPDH*, *BMP2*, *CCDN1*, *CD90*, *COLIA1*, *DSPP*, *OCT4*, *DMP-1*, and *RUNX2* were designed and synthesised by the manufacturer (Microsynth AG, Balgach, Switzerland). The housekeeping *GAPDH* gene served as the endogenous control. Thermocycling conditions were the same as described previously [20]. Expression levels were calculated via the comparative ΔΔCt method (2^−ΔΔCt^ formula) after being normalised to the Ct-value of the GAPDH housekeeping gene. The expression of each gene was presented relative to the reference gene and normalised for the expression of the gene at day 0 in each cell lineage. These experiments were all performed in triplicates, as previously described [20,22,84]. Primer sequences are presented in Table 1.

### 4.8. Statistical Analysis

All experiments were performed in biological and experimental triplicates. The statistical significance level was considered at *p* < 0.05. All statistical analyses of data were performed using GraphPad PRISM 6.0 (GraphPad, La Jolla, CA, USA). Statistical analysis data are presented as average values ± standard deviation for all experiments. Two-way ANOVA tests, followed by the Tukey post hoc test, were used to compare hDPSCs and hBMSCs and time points for each cell type. Asterisks represent statistically significant differences between the various time points and day 0 as control (* *p* value < 0.05; ** *p* value < 0.01; **** *p* value < 0.0001).

## 5. Conclusions

These findings highlight the importance and dynamic nature of Mub-D for dentine and bone tissue repair. Its local application after tooth and bone injury will greatly assist and promote tissue healing. However, additional research should focus on elucidating the molecular mechanisms underlying the effects of Mub-D, particularly its synergistic or antagonistic effects with other molecules involved in tissue regeneration. In vivo studies will definitively validate the therapeutic potential of this synthetic peptide in future clinical settings. Mub-D could stimulate stem cell osteogenic/odontogenic differentiation within teeth and craniofacial bones. Its use in clinics will enhance the regeneration of defective dental and periodontal tissues upon injuries and other pathologic conditions.

## Figures and Tables

**Table 1:** Used primers.

Gene	Accession No.	Forward Primer 5′-3′	Reverse Primer 3′-5′
*GAPDH*	NM_002046.5	AGGGCTGCTTTTAACTCTGGT	CCCCACTTGATTTTGGAGGGA
*BMP2*	NM_001200.2	ATGGATTCGTGGTGGAAGTG	GTGGAGTTCAGATGATCAGC
*CCDN1*	NM_053056.3	GCTGCGAAGTGGAAACCATC	CCTCCTTCTGCACACATTTGAA
*CD90*	NM_006288.3	GAAGGTCCTCTACTTATCCGCC	TGATGCCCTCACACTTGACCAG
*COL1A1*	NM_000088.1	GATTCCCTGGACCTAAAGGTGC	AGCCTCTCCATCTTTGCCAGCA
*DSPP*	NM_014208.3	GCATCCAGGGACCAAGTAAGCA	CTTGGACAACAGCGACATCCT
*OCT4*	NM_001285986.2	CTTTCTCAGGGGGACCAGTG	GGGACCGAGGAGTACAGTGC
*RUNX2*	NG_008020.1	GCCAGGGTCTAGGAGTTGTT	ACCCACCACCCTATTTCCTG
*OSX*	NM_152860.1	CCTCTGCGGGACTCAACAAC	AGCCCATTAGTGCTTGTAAAGG
*DMP-1*	NM_004407.1	GAGCAGTGAGTCATCAGAAGGC	GAGAAGCCACCAGCTAGCCTAT
*OCN*	NM_199173	CGCTACCTGTATCAATGGCTGG	CTCCTGAAAGCCGATGTGGTCA

Abbreviations: *GAPDH*, glyceraldehyde-3-phosphate dehydrogenase; *BMP2*, bone morphogenetic protein 2; *CCDN1*, cyclin D1; *CD90*, cluster of differentiation 90; *COL1A1*, collagen type I alpha 1 chain; *DSPP*, dentin sialophosphoprotein; *OCT4*, octamer binding transcription factor 4; *RUNX2*, runt-related transcription factor 2; *OSX*, osterix (also called transcription factor SP7); *DMP-1*, dentin matrix acidic phosphoprotein 1; *OCN*, osteocalcin.

## Data Availability

Data are contained within the article.
